# Acute Effects of Two Slow-Paced Breathing Protocols With and Without Inspiratory Breath-Holding on Heart Rate Variability: A Randomized Controlled Trial

**DOI:** 10.3390/medsci14060643

**Published:** 2026-10-06

**Authors:** Ana Sedeño-Vidal, Raúl San-Segundo-Riesco, Rafael Lomas-Vega, Alfonso Javier Ibáñez-Vera, Felipe León-Morillas

**Affiliations:** 1 Department of Health Sciences, University of Jaen, Campus las Lagunillas, 23071 Jaen, Spain; 2 Clínica de Fisioterapia Carpo, Calle Altamirano, 34, 28008 Madrid, Spain

**Keywords:** slow paced breathing, breathing exercises, vagus nerve, heart rate variability, autonomic nervous system, parasympathetic nervous system

## Abstract

**Background/Objectives**: Slow-paced breathing (SPB) is associated with changes in heart rate variability, although these responses are strongly influenced by respiratory patterns; the effects of incorporating an inspiratory breath-hold into an SPB protocol remain insufficiently characterized. This study evaluated the immediate effects of two SPB protocols on heart rate variability (HRV) and cervical pressure pain thresholds (PPT). **Methods**: A single-blind, randomized, controlled trial was conducted with 60 healthy adults (aged 18–65) stratified by age and sex, and allocated equally (n = 20 per group) to: Protocol 1 (6 s inhalation, 6 s hold, 6 s exhalation), Protocol 2 (6 s inhalation, 6 s exhalation), or a sham control (general respiratory education). Interventions lasted 5 min. Primary outcomes were time-domain HRV indices, root mean square of successive differences (RMSSD) and standard deviation of normal-to-normal (SDNN), recorded using a Polar H10 chest strap. The secondary outcome was PPT at the fourth cervical vertebra (C4) measured via digital algometry. **Results**: Repeated measures ANOVA revealed a significant time-by-group interaction for heart rate, RMSSD, and SDNN. Both active breathing protocols significantly increased RMSSD and SDNN compared to control, with high effect sizes (η^2^). Notably, Protocol 1 (with inspiratory breath-holding) produced the largest changes in HRV, RMSSD, and SDNN. Conversely, changes in cervical PPT were modest and reached only borderline significance with small effect sizes. **Conclusions**: Both SPB protocols were associated with acute increases in time-domain HRV indices. Protocol 1 (combining inspiratory breath-holding and lower respiratory frequency) produced a larger acute autonomic response, suggesting its potential utility in non-pharmacological interventions for autonomic dysregulation. However, the isolated contribution of breath-holding cannot be separated from the lower respiratory frequency inherent to this protocol.

## 1. Introduction

The diaphragm is composed of three groups of muscle fibers: sternal, costal, and vertebral (or crural) fibers [1] Anatomically, it is a dome-shaped musculotendinous structure formed by two hemidiaphragms joined by the central tendon and traversed by vascular, digestive, sympathetic, and parasympathetic pathways [2,3]. Diaphragmatic contraction generates pressure gradients between the thoracic and abdominal cavities, thereby enabling pulmonary ventilation and effective gas exchange [4,5].

Respiration is a major determinant of beat-to-beat heart period variability. Through its mechanical action, it may contribute to the adaptability and functional integration of these structures, potentially influencing postural regulation [2,6,7]. The diaphragm is the principal inspiratory muscle, whereas its relaxation facilitates expiration. Respiration is a major determinant of beat-to-beat heart period variability through respiratory cardiac interactions, commonly described as respiratory sinus arrhythmia (RSA) [8]. During inspiration, heart period typically shortens, and during expiration, it lengthens, reflecting respiratory modulation of cardiac autonomic control [9]. Importantly, respiratory rate and depth can alter the magnitude of respiratory-related HRV independently of changes in cardiac vagal tone [10].

Heart rate variability (HRV) is defined as the set of parameters reflecting fluctuations in the intervals between consecutive heartbeats, commonly referred to as R–R intervals [11,12]. From a physiological perspective, HRV comprises measures of beat-to-beat variation in cardiac cycle length that are influenced by multiple physiological processes, including autonomic, respiratory, and cardiovascular mechanisms [13,14]. Higher HRV is generally associated with greater physiological flexibility, improved cardiovascular adaptability, and enhanced resilience to stress, whereas lower HRV may reflect impaired adaptive capacity and excessive sympathetic nervous system activation [15].

While slow-paced breathing enhances HRV through cardiorespiratory coupling, the specific physiological contribution of incorporating an inspiratory breath-hold into the breathing cycle remains less understood [14]. It is unknown whether adding an inspiratory hold provides an additive autonomic stimulus, via altered intrathoracic pressure dynamics and baroreflex engagement, or if responses are solely driven by slow breathing. Several studies have reported systemic effects associated with alterations in basal respiratory rhythm; some studies describe improvements in heart rate variability [16], others report beneficial physiological responses in patients with arterial hypertension [17], while additional evidence suggests potential associations with enhanced cognitive performance, increased perceived bodily relaxation, and reduced sympathetic activity [8]. Based on this, the inclusion of an inspiratory breath-hold in slow-paced breathing could produce greater improvement in HRV than slow-paced breathing alone. Thus, the primary aim of this randomized controlled trial was to compare the acute and global effects of two distinct slow-paced breathing protocols on autonomic regulation. The secondary exploratory objective was to examine immediate changes in cervical pressure pain thresholds following the intervention.

## 2. Materials and Methods

### 2.1. Study Design

This study was designed as a single-blind, randomized, controlled clinical trial with a parallel-group design. All procedures were conducted in accordance with the 2025 CONSORT reporting guidelines [18] and complied with the ethical principles of the Declaration of Helsinki. The study protocol was approved by the Institutional Ethics Committee (SICEIA-2025-002737) in December 2025 and was prospectively registered at ClinicalTrials.gov (registration code: NCT07445113).

### 2.2. Participants

Participants were recruited on 27 December 2025 using a convenience sampling method. Consecutive healthy individuals from the University of Jaén were enrolled between 2025 and January 2026. Eligible participants were healthy adults aged 18 to 65 years who did not meet any of the exclusion criteria. The exclusion criteria were as follows: (a) recent trauma or fractures affecting the craniofacial, mandibular, or cervical regions; (b) history of recent abdominal surgery; (c) diagnosed neurological, rheumatic, or systemic diseases; (d) pregnancy; (e) cardiovascular conditions (including arrhythmias or the presence of implanted electronic devices), tumors, or psychiatric disorders under pharmacological treatment; and (f) current use of drugs, alcohol, or medications affecting the central nervous system. All participants were instructed to refrain from smoking and consuming alcohol or caffeine for at least 24 h prior to the experimental session. Each study group consisted of 20 participants. All participants received detailed oral and written information about the study and provided written informed consent prior to enrollment.

### 2.3. Randomization

Randomization was performed using a computer-generated random number sequence created with Microsoft Excel, with a 1:1 allocation ratio to ensure balanced and comparable groups. Participant recruitment followed a consecutive sampling approach, whereby all eligible individuals were included until the required sample size was reached.

To enhance group comparability and homogeneity, an initial stratification by age and sex was performed, generating homogeneous strata for these variables. Within each stratum, participants were randomly assigned to one of the three study groups using permuted block randomization with variable block sizes, thereby maintaining group balance while minimizing predictability of the allocation sequence. The enrolling personnel and the assessor responsible for outcome measurements were blinded to group allocation.

### 2.4. Interventions

A physiotherapist specializing in manual therapy was responsible for administering the interventions across all study groups. All procedures were conducted in a clinical laboratory at the University of Jaén. Participants were placed in a supine position with their hips, knees, and ankles flexed. A pillow was placed beneath their knees to facilitate relaxation of the abdominal musculature. SPB-based intervention consisted of three sequential phases:

- Baseline phase: Participants rested comfortably in a quiet room and breathed spontaneously at their natural respiratory rate.
- Guided breathing phase: Participants performed one of two SPB protocols while receiving real-time auditory guidance. A metronome-like voice dictated the timing of inhalation and exhalation to maintain a consistent respiratory rhythm, ensuring strict adherence to the prescribed patterns throughout the intervention. The session was continuously supervised on a 1:1 basis by the physiotherapist, who visually verified chest and abdominal movements to ensure compliance. Participants were instructed to immediately report dizziness, dyspnea, discomfort, or difficulty maintaining the prescribed breathing pattern. All participants completed the prescribed 5 min intervention without interruption, missed cycles, or recorded adverse events. The protocols were as follows:
  - Protocol 1: 6 s inhalation, 6 s inspiratory hold, and 6 s exhalation.
  - Protocol 2: 6 s inhalation followed by a 6 s exhalation.
- Recovery phase: Participants remained in the supine position and resumed spontaneous breathing.

To ensure an appropriate control condition, the sham intervention was strictly matched in duration and therapist contact time. It consisted of general respiratory education without any guided breathing patterns or rhythm modulation. The guided breathing intervention lasted 5 min and was standardized across groups.

### 2.5. Outcome Measures

All assessments were conducted by an experienced physiotherapist. Outcome measures were collected at baseline (T1, pre-intervention) and immediately after completion of the breathing intervention (T2, post-intervention).

#### 2.5.1. Primary Outcome: Heart Rate Variability

Heart rate variability (HRV) was assessed using a Polar H10 [19,20] chest strap (Polar Electro Oy, Kempele, Finland) and the Elite HRV mobile application (Elite HRV Inc., Asheville, NC, USA) [21], which served strictly as the data acquisition interface to export raw RR interval series. This variable is expressed in arbitrary units on a 0–100 scale and is computed using a proprietary algorithm based on the natural logarithmic transformation of RMSSD (In RMSSD) to evaluate overall autonomic readiness and modulation [22]. Participants were placed in a supine position with the head supported on a pillow. All measurements were conducted in the same standardized supine position. Following a 5 min accommodation period, HRV was continuously recorded for 5 min using a Polar H10 chest-strap heart rate monitor (Polar Electro Oy, Kempele, Finland) and the Elite HRV mobile application. RR interval data were subsequently exported and processed using Kubios HRV Standard version 3.5.1 (Kubios Oy, Kuopio, Finland). The RR interval series were automatically processed for artifact detection and correction using Kubios HRV. The complete 5 min recording was used as the analysis epoch for the calculation of HRV indices [23].

The following time-domain HRV parameters were extracted for analysis: the root mean square of successive differences (HRV-RMSSD) and the standard deviation of normal-to-normal interbeat intervals (HRV-SDNN) [24]. HRV-RMSSD was used as a time-domain index of short-term HRV, with substantial sensitivity to respiratory-related cardiac variability. Given the respiratory manipulation used in the present study, RMSSD values were interpreted as an HRV measure rather than as a direct measure of cardiac vagal tone, whereas HRV-SDNN was considered as a measure of overall variability in normal-to-normal heart periods during the recording period and overall autonomic adaptability, commonly associated with metabolic and physiological flexibility [25,26].

#### 2.5.2. Secondary Outcomes

Pressure pain sensitivity was assessed using a digital pressure algometer (FPIX 50, Wagner Instruments, Greenwich, CT, USA) to determine pressure pain thresholds (PPT), defined as the minimum pressure required to elicit pain or discomfort. PPT measurements were obtained at the spinous process of the fourth cervical vertebra (C4). For C4 assessment, participants were seated upright, and pressure was applied perpendicularly to the spinous process. The evaluator gradually increased the applied pressure and instructed participants to indicate the moment at which the sensation became uncomfortable or painful. Two measurements were obtained at each site, separated by an interval of approximately ±20 s, and the mean of both measurements was used for subsequent analysis.

Heart rate data were recorded using the Polar H10 [19,20] chest strap (Polar Electro Oy, Kempele, Finland) and the Elite HRV mobile application (Elite HRV Inc., Asheville, NC, USA) [21]. Mean heart rate (HR) was obtained from the same RR interval recordings collected with the Polar H10 device during the same measurement periods. Resting HR was measured before and after the experimental protocol using the Polar H10 device. Although no harm was expected, all participants were asked about any adverse events after the intervention and were advised to stay in the research facilities for 15 min before leaving.

### 2.6. Sample Size Calculation

Sample size estimation was performed using G*Power software, version 3.1 (Düsseldorf, Germany), based on previous studies employing similar outcome variables, specifically RMSSD, using data reported by Prinsloo et al. [27] (2013). Assuming a confidence level of 95% and a statistical power of 90%, and considering an expected effect size of Cohen’s f = 0.25 for differences among three groups evaluated using repeated-measures ANOVA within-between interaction, a minimum of 18 participants per group was required, resulting in a total sample size of 54 participants. Accounting for an anticipated dropout rate of approximately 10%, a total of 60 participants distributed across the three treatment arms was deemed necessary.

### 2.7. Data Analysis

The data were collected on paper and using the computer applications available for the electronic measurement instruments. Both the data on the paper forms and the data from the applications were digitized and transferred to a spreadsheet (Microsoft Excel). There, the data were organized into rows representing one participant and columns containing the data for each variable. These data were then tabulated, and its integrity and accuracy were verified.

Subsequently, the data were transferred to the Statistical Package for the Social Sciences (IBM Corp., released 2015, IBM SPSS Statistics for Windows, Version 23.0. Armonk, NY, USA: IBM Corp.). This software was used for calculating and storing secondary variables, primarily for obtaining scores on some questionnaires and calculating change scores, as well as for conducting the inferential analyses. This new information was transferred to the MedCalc^®^ Statistical Software version 23.4.8 (MedCalc Software Ltd., Ostend, Belgium; https://www.medcalc.org; 2026). This software was used to generate the results graphs.

Data description was performed by calculating arithmetic means and standard deviations for quantitative variables, both continuous and discrete. Frequencies and percentages were used for categorical variables. The Kolmogorov–Smirnov test was used to test the normality of the data, and Levene’s test was used to test the equality of variances or homoscedasticity.

Baseline comparability between groups was analyzed using one-way analysis of variance. Inferential analysis was performed using repeated measures analysis of variance (ANOVA) with the factors of time (pre- and post-intervention scores) and treatment group. The hypothesis of interest was the time-group interaction. Post hoc comparisons were performed using Dunnett’s T3 test, which is particularly suitable for small samples (n < 50) and is highly robust to a lack of homoscedasticity. Effect size was measured with partial eta-squared (η^2^p), which is the equivalent of the coefficient of determination (R^2^) for experimental studies and could be interpreted as the proportion of between-group differences due to treatment. According to Cohen [28], an η^2^ < 0.02 can be considered insignificant, small if it is between 0.02 and 0.15, medium if it is between 0.15 and 0.35, and large if it is >0.35.

## 3. Results

**Figure 1:**
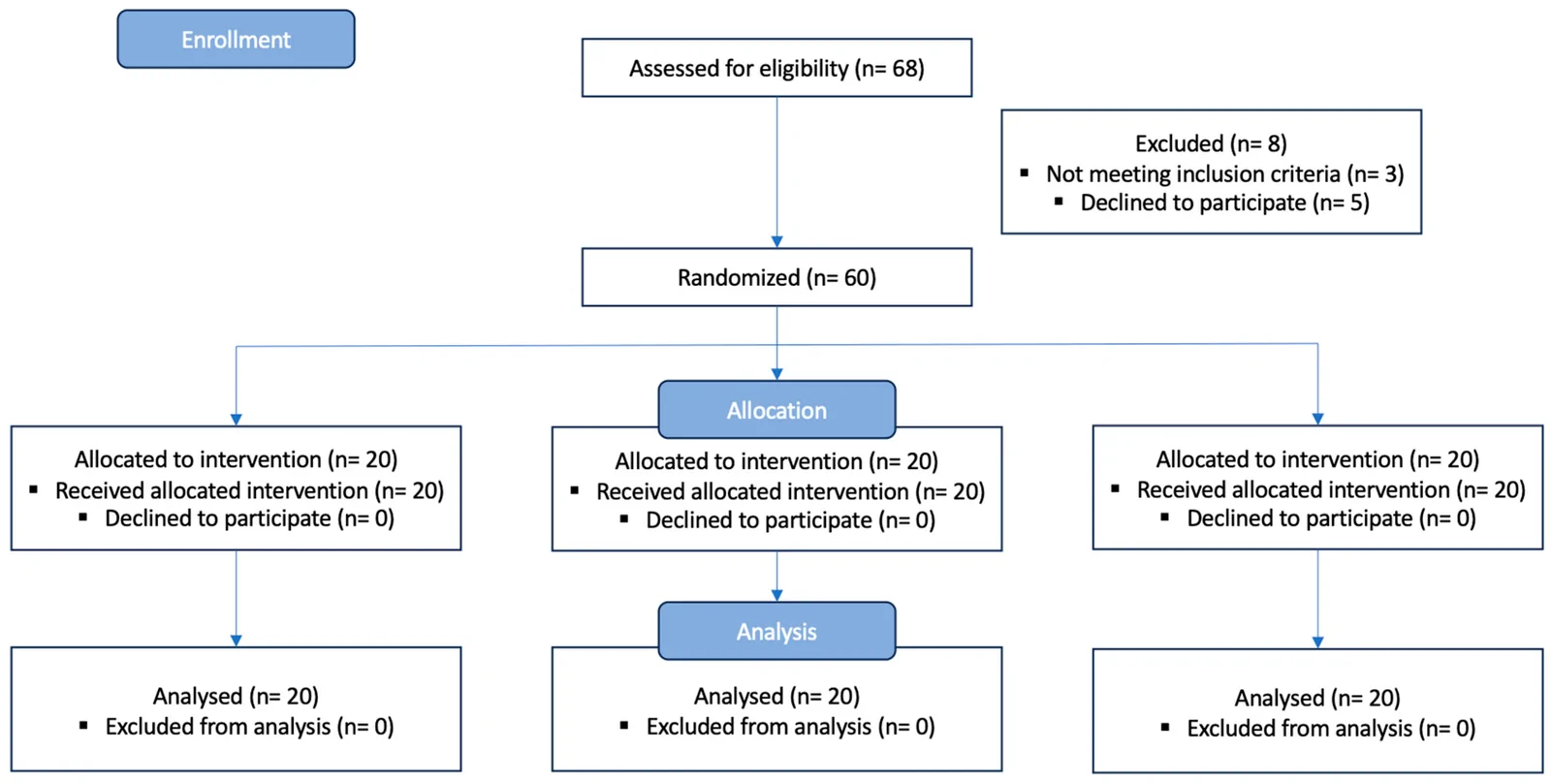
Flow diagram of group allocation.

Finally, 20 subjects were assigned to each group (Figure 1). Gender allocation resulted in a balanced distribution of 10 female and 10 male subjects in each treatment group. One-way analysis of variance indicated no significant baseline differences for the main prognostic variables (Table 1). The applicability of repeated-measures ANOVA was verified by checking the normality of the distribution of all samples and confirming compliance with Mauchly’s sphericity criterion. However, Levene’s test confirmed a violation of the assumption of equality of error variances; consequently, the use of Dunnett’s T3 test was deemed appropriate for the post hoc comparisons.

Repeated-measures ANOVA indicated a time-by-group interaction for the variables HR, HRV, RMSSD, and SDNN (Table 2). The interaction was borderline significant for algometry. The effect size was considered small for Algometry, medium for HR, and large for the rest of the variables analyzed. No harms were reported by participants.

**Figure 2:**
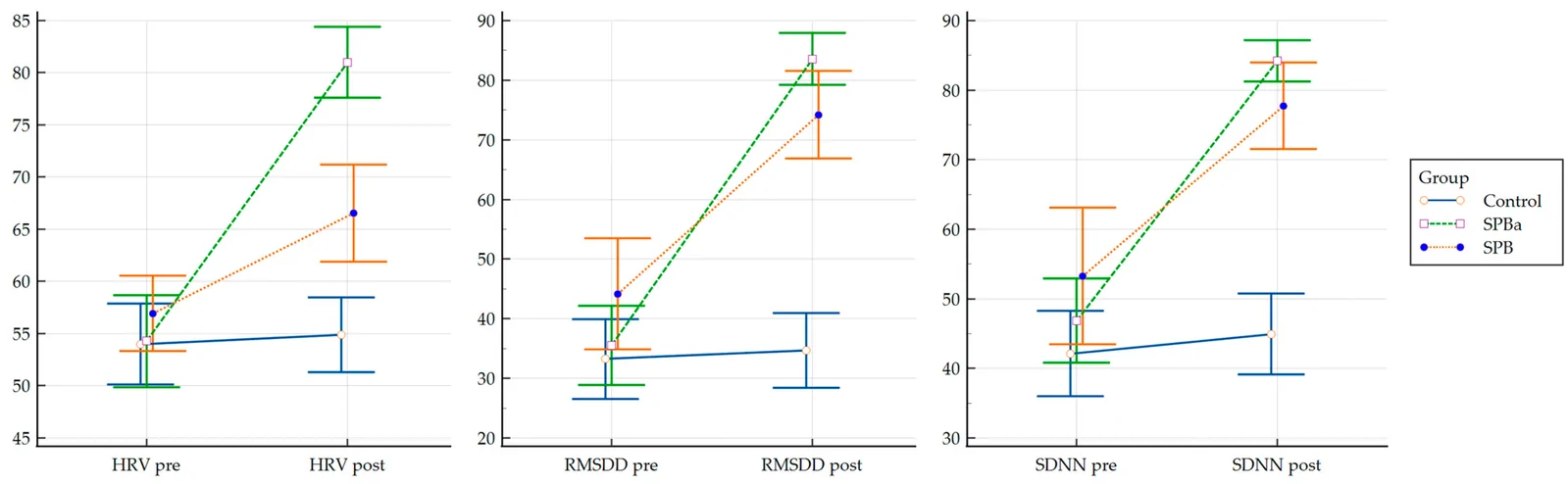
Between-group mean differences.

After performing the post hoc comparisons, Dunnett’s T3 test revealed significant differences in comparisons between the control and treatment groups for HRV, RMSSD, and SDNN variables. However, for HR, a difference at the level of statistical significance was found only between the control group and the SPB group. Details of this analysis are shown in Table 3, and the results are represented graphically in Figure 2.

## 4. Discussion

The present randomized controlled trial investigated the immediate effects of two SPB protocols on autonomic modulation and cervical pressure pain sensitivity in healthy adults. The main finding was that both breathing interventions were associated with significant changes in time-domain HRV indices, as reflected by improvements in HRV-related parameters, whereas the protocol incorporating inspiratory breath-holding demonstrated the largest changes in RMSSD and SDNN. In contrast, changes in cervical PPT were small. Together, these results suggest that paced breathing was associated with acute changes in cardiac beat-to-beat variability, with Protocol 1 (breath-holding plus lower respiratory rate) showing the largest magnitude of response. However, this enhancement reflects the combined effect of both structural components rather than breath-holding alone.

The observed autonomic effects are consistent with prior evidence demonstrating that slow breathing has been associated with increases in HRV indices. Breathing at lower frequencies has been shown to increase HRV, particularly when respiratory rhythm approaches ranges that optimize cardiorespiratory coupling and respiratory–cardiovascular synchronization [29]. Previous work on paced and diaphragmatic breathing has similarly reported increases in vagally mediated indices such as RMSSD, alongside broader improvements in autonomic flexibility [30,31,32]. Zaccaro et al. [8] further highlighted that slow breathing induces transient changes in time-domain HRV indices through coordinated respiratory–cardiovascular interactions, with additional effects on emotional and cognitive processes. Recently, Shaffer et al. [32] emphasized paced breathing as a simple and effective strategy to modulate HRV via vagal mechanisms.

While several physiological mechanisms—such as baroreflex sensitivity and mechanical intrathoracic pressure changes—could theoretically underlie the observed changes, these underlying pathways were not directly quantified in the present study [33]. Therefore, discussions regarding cardiorespiratory coupling remain hypothetical explanations supported by prior physiological literature rather than direct observational findings of this trial.

Slower respiratory cycles increase RSA by prolonging the inspiratory-expiratory phases, which triggers the baroreflex in response to rapid changes in blood pressure detected by baroreceptors. This, in turn, regulates heart rate and vascular tone, resulting in greater coupling between blood pressure and heart rate variability. Together, these mechanisms provide a plausible explanation for the increases in RMSSD and SDNN observed after both interventions [34].

A key finding of the present study was that the protocol incorporating an inspiratory breath-hold produced larger changes in RMSSD and SDNN than the other breathing protocol. When interpreting these physiological differences, it must be emphasized that Protocol 1 and Protocol 2 differed in two concurrent dimensions. However, these differences cannot be attributed specifically to breath-holding because the protocol also involved a lower respiratory frequency. Consequently, the present design does not allow the independent contribution of inspiratory breath-holding to be isolated. Although both interventions improved HRV indices, the addition of inspiratory breath-holding consistently produced larger effects, suggesting a potential role for breath-hold components in amplifying acute HRV responses. However, empirical evidence on inspiratory breath-holding embedded within structured breathing protocols remains limited, and the underlying mechanisms are not yet well established [35].

One possible explanation relates to the respiratory–hemodynamic consequences of inspiratory breath-holding and lower breathing frequency. Speculatively, breathing at lower frequencies may engage different baroreflex resonance dynamics compared to frequencies in Protocol 2, operating alongside the intrathoracic pressure shifts induced by temporary airflow cessation. Prolonging the post-inspiratory phase could alter intrathoracic pressure dynamics and venous return, potentially influencing baroreflex engagement. However, as continuous hemodynamic and baroreflex parameters were not recorded, such physiological mechanisms require direct confirmation in future mechanistic studies. In addition, the extension of the respiratory cycle inherently increases the time available for RSA expression, which may further contribute to the observed HRV enhancement [35,36,37]. Moreover, prolonged inspiratory phases may improve ventilation distribution and alveolar recruitment, potentially optimizing gas-exchange efficiency and stabilizing tidal-volume dynamics. Together, these effects may reinforce respiratory–cardiovascular coupling and contribute to the greater autonomic response observed in the breath-holding condition [38]. Nevertheless, the heterogeneity of protocols across studies, including differences in respiratory cadence, inspiratory-to-expiratory ratios, and intervention duration, limits direct comparison. It is important to note that few studies have specifically isolated inspiratory breath-holding as an integral component of slow-breathing interventions, making the present findings a significant contribution to the existing literature. These findings provide physiological evidence that adding inspiratory breath-holding to slow-paced breathing enhances acute autonomic responsiveness in young healthy adults. However, as the sample consisted of young participants (mean age 21–22 years) undergoing a single 5 min intervention, these findings cannot be directly extrapolated to older populations or clinical cohorts with baseline autonomic dysfunction. Modulating cardiorespiratory coupling through slow-breathing protocols could support non-pharmacological strategies aimed at acutely influencing cardiac autonomic responsiveness.

In contrast, changes in cervical pressure pain sensitivity were limited. Although a marginal interaction was observed for pressure pain thresholds (PPTs) at the C4 spinous process, overall effect sizes were small. These findings differ from previous evidence in clinical populations linking autonomic function to nociceptive modulation. For instance, reduced heart rate variability has been associated with heightened pain sensitivity and chronic neck pain [39,40], whereas enhanced baroreflex activation has been shown to inhibit nociceptive transmission [40]. Furthermore, Falla et al. [41] reported altered breathing patterns and sympathetic hyperactivity in patients with chronic neck pain, suggesting that interventions targeting cardiorespiratory regulation may hold therapeutic relevance.

Within this context, the absence of significant changes in our healthy sample likely reflects the relatively stable nociceptive profile of these individuals, characterized by the absence of baseline autonomic dysfunction or central sensitization. In addition, acute HRV parameters following a single intervention session may be insufficient to induce immediate changes in central pain-processing pathways [42]. However, this mechanistic interpretation should be considered in light of an additional methodological limitation: the inspiratory breath-holding protocol resulted in a lower effective respiratory frequency than conventional SPB. Consequently, the present findings do not allow disentangling the specific contribution of breath-hold components from the effects attributable to reduced breathing frequency *per se*.

HRV is widely recognized as an index of autonomic flexibility and physiological adaptability [43]. However, it is influenced by multiple interacting factors, including sleep quality, physical activity, psychological stress, circadian variation, and breathing behavior. Accordingly, the increases observed in RMSSD and SDNN should be interpreted as short-term markers of autonomic responsiveness to controlled respiratory modulation rather than stable adaptations.

Several limitations should be acknowledged. First, respiratory airflow or respiratory volume was not continuously recorded; therefore, adherence to the prescribed respiratory pattern could not be physiologically verified, and the contribution of respiratory variability to the observed HRV changes cannot be quantified. Thus, future studies must consider the inclusion of a respiratory belt or complementary tools for monitoring respiration. Second, a methodological limitation lies in the design of the active protocols, which differed simultaneously regarding the presence of inspiratory apnea and effective respiratory rate (Protocol 1 versus Protocol 2). Given that a lower respiratory rate is known to increase HRV parameters, the autonomic changes observed in Protocol 1 reflect the combined and synergistic effect of both factors rather than the isolated contribution of inspiratory apnea. Consequently, it is not possible to disentangle these design characteristics; therefore, future studies employing protocols with matched respiratory rates are required to isolate the independent physiological effect of apnea. Third, no subjective measures of perceived breathing effort, discomfort, relaxation, or affective state were collected, limiting interpretation of the subjective experience of the intervention. Fourth, cervical PPT was a secondary exploratory outcome in a healthy sample and may have been subject to restricted variability or ceiling effects. Finally, only immediate post-intervention responses were assessed, and the findings cannot be generalized to clinical populations or interpreted as evidence of sustained physiological adaptation.

Future studies should determine whether repeated exposure to these protocols induces sustained autonomic adaptations and whether such changes translate into clinically meaningful outcomes.

## 5. Conclusions

In conclusion, both slow breathing interventions elicited immediate improvements in autonomic regulation, with the inspiratory breath-holding protocol producing the strongest effects. These findings support paced breathing as an effective acute modulator of HRV in healthy adults, though the response in Protocol 1 reflects the synergistic effect of breath-holding and reduced breathing frequency. However, because changes in respiratory rate and breath-holding occurred concurrently, future studies employing frequency-matched protocols are required to definitively isolate the independent physiological contribution of inspiratory breath-holding.

## Figures and Tables

**Table 1:** Baseline comparability of the groups.

	Control	(n= 20)	SPBa	(n = 20)	SPB	(n = 20)	ANOVA
	Mean	(SD)	Mean	(SD)	Mean	(SD)	*p*-Value
Age	21.10	(0.31)	21.85	(1.53)	21.75	(1.37)	0.109
Height	1.71	(0.10)	1.70	(0.11)	1.70	(0.10)	0.903
Weight	67.80	(10.88)	66.34	(11.86)	66.81	(10.63)	0.914
BMI	22.90	(1.20)	22.68	(1.43)	22.89	(1.19)	0.833
HR	73.95	(7.22)	72.95	(11.90)	69.60	(7.39)	0.293
HRV	54.00	(8.32)	54.30	(9.43)	56.95	(7.69)	0.487
RMSSD	33.26	(14.23)	35.51	(14.22)	44.18	(19.88)	0.091
SDNN	42.14	(13.17)	46.87	(12.93)	53.29	(20.95)	0.099
ALGOM	5.11	(0.34)	5.06	(0.50)	5.14	(0.36)	0.818

BMI: body mass index; HR: heart rate; HRV: heart rate variability; RMSSD: root mean square of successive differences; SDNN: standard deviation of normal-to-normal; ALGOM: algometry; n: sample; SPBa: protocol 1 of slow-paced breathing; SPB: protocol 2 of slow-paced breathing; SD: standard deviation.

**Table 2:** Mean scores before and after the intervention. *p*-Value and effect size of the time-by-group interaction.

	Control		SPBa		SPB		Time-by-Group Interaction	
	Mean	(SD)	Mean	(SD)	Mean	(SD)	*p*-Value	η^2^p
HR post	74.05	(7.52)	69.50	(11.83)	67.25	(7.81)	0.008	0.154
HRV post	54.90	(7.67)	81.00	(7.25)	66.55	(9.94)	<0.001	0.646
RMSSD post	34.71	(13.44)	83.59	(9.30)	74.23	(15.64)	<0.001	0.843
SDNN post	44.95	(12.42)	84.23	(6.36)	77.74	(13.30)	<0.001	0.690
ALGOM post	5.19	(0.25)	5.25	(0.47)	5.22	(0.31)	0.050	0.099

HR post: heart rate after intervention; HRV post: heart rate variability after intervention; RMSSD post: root mean square of successive differences after intervention; SDNN post: standard deviation of normal-to-normal intervals after intervention; ALGOM post: algometry after intervention; SPBa: protocol 1 of slow-paced breathing; SPB: protocol 2 of slow-paced breathing; SD: standard deviation; η^2^p: partial Eta-squared.

**Table 3:** Between-group differences in change scores compared to the control group.

Variable	Comparison Group	Differences in Change Scores	Standard Error	*p*-Value	95% Confidence Interval	
					Upper Bound	Lower Bound
HR	SPBa	2.77	3.06	0.744	−4.91	10.46
	SPB	5.57	2.33	0.063	−0.23	11.38
HRV	SPBa	−13.20	2.32	<0.001	−18.98	−7.42
	SPB	−7.30	2.41	0.013	−13.32	−1.28
RMSDD	SPBa	−25.57	3.89	<0.001	−35.30	−15.84
	SPB	−25.23	4.93	<0.001	−37.54	−12.91
SDNN	SPBa	−22.01	3.47	<0.001	−30.70	−13.32
	SPB	−21.97	4.60	<0.001	−33.47	−10.48
ALGOM	SPBa	0.00	0.12	1.000	−0.32	0.31
	SPB	−0.03	0.10	0.990	−0.27	0.22

HR: heart rate; HRV: heart rate variability; RMSSD: root mean square of successive differences; SDNN: standard deviation of normal-to-normal; ALGOM: algometry; SPBa: protocol 1 of slow-paced breathing; SPB: protocol 2 of slow-paced breathing.

## Data Availability

The dataset is available on request from the authors.

## Abbreviations

The following abbreviations are used in this manuscript:

HRV	Heart rate variability
RMSSD	Root mean square of successive differences
SDNN	Standard deviation of normal-to-normal
ANS	Autonomic nervous system
